# The Uniqueness of 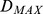-Matrix Graph Invariants

**DOI:** 10.1371/journal.pone.0083868

**Published:** 2014-01-02

**Authors:** Matthias Dehmer, Yongtang Shi

**Affiliations:** 1 Institute for Bioinformatics and Translational Research, UMIT, Eduard Wallnoefer Zentrum 1, A-6060, Hall in Tyrol, Austria; 2 Center for Combinatorics and LPMC-TJKLC, Nankai University, Tianjin, P.R. China; Queen's University Belfast, United Kingdom

## Abstract

In this paper, we examine the uniqueness (discrimination power) of a newly proposed graph invariant based on the matrix 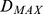 defined by Randić et al. In order to do so, we use exhaustively generated graphs instead of special graph classes such as trees only. Using these graph classes allow us to generalize the findings towards complex networks as they usually do not possess any structural constraints. We obtain that the uniqueness of this newly proposed graph invariant is approximately as low as the uniqueness of the Balaban 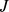 index on exhaustively generated (general) graphs.

## Introduction

Matrix-based descriptors have been developed extensively [1]–[3]. As a result, the distance matrix, the adjacency matrix and other graph-theoretical matrices [4] have been used to define topological graph measures and to examine their properties [4], [5]. A property which has been of considerable interest when designing topological descriptors is referred to as uniqueness [6]–[8]. Generally, the uniqueness of a structural graph measure relates to the ability to distinguish the structure of non-isomorphic graphs uniquely. From a mathematical point of view, the low uniqueness or high degeneracy of a graph measure under consideration is an undesired aspect as non-isomorphic graphs should be mapped to non-equal values. Such a highly discriminating graph invariant could be then used to distinguish graph structures uniquely and, thus, to perform graph isomorphism testing [9], [10]. In the context of graph isomorphism testing, so-called complete graph invariants have been investigated [9], [11]. Such a graph invariant has the property that it discriminates all non-isomorphic graphs uniquely (i.e., without any degeneracy) and isomorphic graphs are mapped to equal values [9], [11]. For example, Liu and Klein [11] made an attempt to derive complete graph invariants by using eigenvalues. Dehmer et al. [8], [9] defined graph entropies which turned out to be the most discriminative measures so far when using exhaustively generated graphs. Clearly, such measures are suitable candidates to test graph isomorphism efficiently [9].

Recently, Randić et al. [2], [12] defined so-called 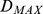 matrices and also topological descriptors thereof. Let 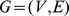 be a finite graph. Then these matrices have been defined by using the ordinary distance matrix 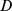 of 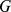 such that in each row and column the dominant (largest) distances are used where other elements 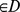 are set to be zero, see [2], [12]. Moreover they defined a new topological index 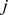 which has the same definition than the well-known Balaban index 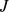 [13] but uses 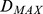 instead of only using 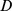. Then based on example claculations, Randić et al. [2] argued that 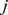 may be a promising candidate for isomorphism testing, but they did not examine the problem in depth on wider classes of graphs.

In this paper, we explore the uniqueness of 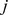 by employing 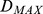 on a large scale. For this, we use exhaustively generated graphs with 9 and 10 vertices each [8] and alkane trees 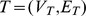 where 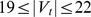. Our findings (see section 'Methods and Results') reveal that the uniqueness of 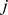 is always worse than the one of 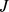 and, thus, the uniqueness of 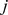 is insufficient for performing isomorphism testing.

## Methods and Results

### The Structural Descriptors 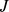 and 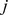


Let 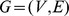 be a finite graph. To define the Balaban index 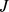 [13], [14] of 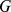, let 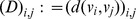 be the distance matrix. 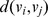 is the topological distance between 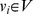 and 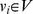. For each vertex 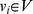, 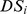 denotes the distance sum (row or column sum) by adding the entries in the corresponding row or column of 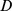. Let 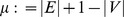 be the cyclomatic number [14]. Then 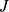 has been defined by [13]

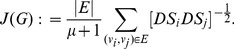
(1)


A critical analysis to examine the uniqueness of 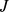 and other quantities has recently been carried out by Dehmer et al. [8] based on using exhaustively generated (general) graphs. In this sense Dehmer et al. examined the limitations of the Balaban 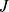 index and found that this index is quite unstable [9]; here that means there is a strong dependency between the sample size of the graph set and the uniqueness [8]. To study the technical details and the precise definitions, we refer to [8], [9]. Moreover, the findings of Dehmer et al. [8] revealed that the uniqueness of 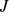 by using exhaustively generated graphs is poor. For example by using the class 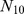 (all non-isomorphic graphs with 10 vertices), 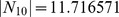, the Balaban index 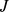 could only discriminate 20% of 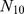 uniquely. Nevertheless, 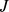 has high uniqueness for alkane trees and isomers [8], [9], [13].

To define 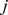, we require the definition of 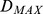 [12]: 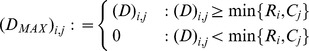



Following Randić et al., the topological index 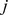 is just the 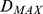 analog to Balaban's 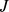 index, see [2]. Based on the fact that 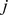 can discriminate the remaining isomers of 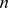-dodecane and 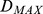 has often a different structure compared to 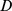, Randić et al. concluded that 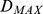 and, hence, 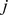 may be a promising tool for graph isomorphism testing, see [2]. In the next section, we see that this statement has been too premature when evaluating 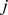 on general and exhaustively generated graphs. By evaluating characteristic properties (e.g., the uniqueness) of topological graph measures on such (general) graphs, one can conclude how would the index behave in the context of using complex networks.

## Results

Before interpreting Table 1, we explain its notation. We here used the graph classes 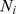, 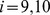 [8] and 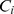, 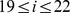 [8]. Again 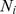 is the class of all exhaustively generated non-isomorphic and connected graphs with 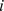 vertices [8]. 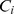 is the class of exhaustively generated non-isomorphic and connected alkane trees [8]. ndv stands for the number of non-distinguishable values [8] and 
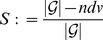
 where 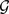 is a class of graphs, see [7].

**Table 1:** The uniqueness of 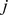 and 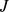 measured by ndv and 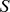.

Uniqueness of 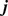 and 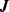				
	Graph class	cardinality	ndv	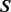
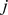 based on 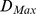	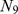	261080	165109	0.36759
	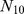	11716571	9476268	0.1912081
	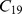	148284	144041	0.028614
	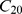	366319	359177	0.019496
	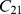	910726	898838	0.013053
	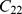	2278658	2258804	0.00871302
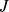	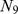	261080	156674	0.399900
	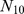	11716571	9307263	0.205633
	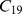	148284	5967	0.95975
	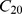	366319	44800	0.877702
	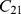	910726	45703	0.949816
	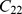	2278658	306911	0.865310

Table 1 shows numerical results when comparing 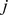 and 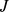 on the just explained graph classes. We observe that the uniqueness of 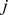 is quite poor for all graph classes. In case of using 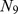, the uniqueness of 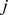 is approximately as low as the uniqueness of 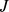. That means both topological indices can only discriminate about 39% out of 261080 graphs. By considering the results for 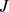, we see that 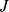 possesses high uniqueness when using 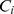. Note that this has already been found by Balaban [13] and Dehmer et al. [8]. But it is surprising that the uniqueness of 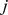 is, without exception, much worse than the one of 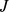. Table 2 shows that 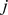 can discriminate the isomers of 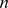-dodecane for which the Balaban 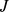 index is pairwisely degenerated.

**Table 2:** The values of 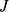 and 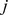 by using the isomers of n-dodecane.

Graph ID	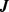	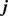
1	4.252509	13.15875
2	4.252509	16.04085
3	3.752273	15.86058
4	3.752273	11.99851
5	4.135003	15.04953
6	4.135003	11.07033
7	3.575256	13.16886
8	3.575256	11.81169
9	3.773441	21.73837
10	3.773441	7.43385
11	3.954123	12.76649
12	3.954123	10.84469

A hypothesis is that the sparseness of 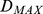 leads to this effect described above. So this matrix can not capture the complexity of the used graphs meaningfully and, thus, 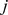 is degenerated for most of the graphs. This result shows the complexity of the problem to construct highly unique graph measures on general and exhaustively generated graphs.

## Summary and Conclusion

This paper investigated the uniqueness of the recently developed topological index 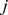 introduced by Randić et al. [2]. 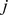 has been defined quite similarly as it is based on the novel matrix 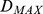 instead of 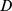. Based on small tests and by only using example graphs, Randić et al. [2] hypothesized 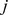 has higher uniqueness than 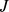 and, the index 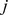 which combined with index 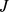, may suffice to resolve the graph isomorphism issue for most cases of molecular graphs.

In this paper we have evaluated this hypothesis on a large scale by using general graphs. In fact, our study disproved this conjecture and demonstrated that the uniqueness of 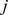 is quite poor by using general exhaustively generated graphs and alkane trees. As future work, we plan to determine so-called degeneracy classes analytically for performing a proper mathematical treatment of the problem. In any way, the search for highly discriminating graph invariants should be continued [8], [9], [15], [16]. Following Randić et al. [2], such measures could be used as a prescreening method and would eliminate need for detailed and elaborate tests on large number of cases. Also, this fact has already been raised by Dehmer et al. [9] where they developed information-theoretic network measures with very low degeneracy on exhaustively generated graphs for graph isomorphism testing.
